# Draft Genome Sequence of *Mycobacterium tuberculosis* Strain MT43, a Representative of the Manu2 Genotype

**DOI:** 10.1128/genomeA.00579-15

**Published:** 2015-06-04

**Authors:** Djaltou Aboubaker Osman, Michael Phelippeau, Didier Musso, Catherine Robert, Caroline Michelle, Olivier Croce, Michel Drancourt

**Affiliations:** a Aix Marseille Université, URMITE, UMR CNRS 7278, IRD 198, INSERM 1095, Marseille, France; b Institut de Recherche Médicinale (IRM), Centre d’Études et de Recherche de Djibouti (CERD), Djibouti, Djibouti; c Institut Louis Malardé, Papeete, French Polynesia

## Abstract

We announce the draft genome sequence of *Mycobacterium tuberculosis* strain MT43, isolated from a pulmonary form of tuberculosis in French Polynesia. Analyzing its 4,145,007-bp, 65.17% G+C chromosome confirmed a fully antibiotic-susceptible Manu2 spoligotype.

## GENOME ANNOUNCEMENT

*Mycobacterium tuberculosis* strain MT43 was isolated from a female 56-year-old French Polynesian patient suffering from pulmonary and pleural tuberculosis (D. Aboubaker Osman, M. Phelippeau, M. Drancourt, and D. Musso, unpublished data). Spoligotyping indicated the spoligotype international type SIT1634 (Manu2), which is one “ancestral” lineage of *M. tuberculosis* comprising only two isolates from the United States, one from Indonesia, and one from Taiwan in the SITVIT database. Recently, at least nine isolates belonging to SIT1634 were described in China, and this spoligotype has been reported to be associated with isoniazid susceptibility testing discrepancies (1). Therefore, we thought that analyzing the whole-genome sequence of *M. tuberculosis* MT43 could help to determine the phylogenetic relationships within the *M. tuberculosis* complex and assess for genetic determinants of antibiotic resistance.

Genomic DNA extracted from *M. tuberculosis* MT43 grown in MGIT Middlebrook liquid culture (Becton Dickinson, Le Pont-de-Claix, France) at 37°C by the cetyl-trimethylammoniumbromide method (2) was sequenced on the Illumina MiSeq platform throughout three runs using 5-kb mate-pair libraries in a 2 × 250-bp run for each barcoded library. The whole set of reads was trimmed using Trimmomatic (3) and assembled with the assembler software Spades (4, 5). Contigs were combined together by SSPACE (6) and Opera (7), with help from GapFiller (8), and refined with homemade tools in Python. Finally, the draft genome of *M. tuberculosis* MT43 strain consists of 15 contigs without gap for a total of 4,145,007 bp and a 65.17% G+C content. Noncoding genes and miscellaneous features were predicted using RNAmmer (9), ARAGORN (10), Rfam (11), Pfam (12), and Infernal (13). Coding DNA sequences (CDSs) were predicted using Prodigal (14), and functional annotation was achieved using BLAST+ (15) and HMMER3 (16) against the UniProtKB database (17).

The genome was shown to encode at least 76 predicted RNAs, including 3 rRNAs, 45 tRNAs, 1 tmRNA, and 27 miscellaneous RNAs. A total of 2,786 genes spanning over 3,722,052 bp were also identified, representing a coding percentage of 89.8%. Among these genes, 274 (6.88%) were assigned as putative proteins and 560 (14.05%) were assigned as hypothetical proteins. Moreover, 2,786 genes matched at least one sequence in the Clusters of Orthologous Groups (COGs) database (18, 19) with BLASTp default parameters. Genomic analysis confirmed a Manu2 spoligotype. To identify known drug-resistance markers, we used the online TB profiler database (http://tbdr.lshtm.ac.uk); no SNPs conferring resistance were found, confirming the *in vitro* data that *M. tuberculosis* strain MT43 is fully susceptible to antibiotics.

### Nucleotide sequence accession numbers.

The genome sequence of *M. tuberculosis* strain MT43 has been deposited with its annotations at EMBL under the accession numbers CVMY01000001 to CVMY01000015.
